# HspX promotes the polar localization of mycobacterial protein aggregates

**DOI:** 10.1038/s41598-019-51132-w

**Published:** 2019-10-10

**Authors:** Yi-Wei Zhang, Jun-Hao Zhu, Zhen-Qi Wang, You Wu, Xianbin Meng, Xuhui Zheng, Babak Javid

**Affiliations:** 1 Centre for Global Health and Infectious Diseases, Collaborative Innovation Centre for the Diagnosis and Treatment of Infectious Diseases, Tsinghua University School of Medicine, Beijing, China; 2 School of Life Sciences, Peking University, Beijing, China; 3 College of Biological Sciences, China Agricultural University, Beijing, China; 4 MOE Key Laboratory of Bioinformatics, School of Life Sciences, Tsinghua University, Beijing, China; 5 Present Address: Immunology and Infectious Diseases, Harvard TH Chan School of Public Health, Boston, USA; 6 Beijing Advanced Innovation Center for Structural Biology, Beijing, China

**Keywords:** Protein aggregation, Bacteriology

## Abstract

Misfolding of translated proteins occurs in all domains of life. In most cells, misfolded proteins coalesce in discrete aggregates at distinct cellular locations. In many bacteria, including mycobacteria, protein aggregates are located at the cellular pole. Yet the mechanism by which aggregates are sorted to the mycobacterial pole is not known. Here, we show that in *Mycobacterium smegmatis*, the small heat shock protein HspX plays a critical role in the polar localization of aggregates of a model fluorescent misfolded protein, GLR103. HspX itself has a polar localization, which is dependent on its N-terminal domain. In a strain deleted for *hspX*, GLR103 is less liable to aggregation and no longer localizes to the pole, and redirecting HspX to the septum radically disrupts the normal polar localization of GLR103 aggregates. To further investigate the role of HspX in native protein aggregation, we performed semi-quantitative mass-spectrometry of mycobacterial protein aggregates in wild-type, *hspX*-deleted and *hspX*-overexpressing strains. We identified a subset of proteins that appeared to be HspX-dependent for aggregate formation. Furthermore, we demonstrate that for validated native protein aggregates, sorting to the cellular pole following proteotoxic stress required HspX. In summary, we have identified the cellular function of HspX in *Mycobacterium smegmatis* as both a pro-aggregase and polar sortase.

## Introduction

Protein misfolding is an inevitable consequence of protein synthesis, and thus all cells have evolved mechanisms to deal with misfolded proteins. Protein homeostasis, proteostasis, involves a complex interplay between rates of gene translation, protein misfolding, refolding and degradation^1^. Refolding of misfolded proteins is mostly performed by large heat shock proteins (HSPs) such as HSP60, HSP70 and HSP100 in an ATP-dependent manner^2^. Misfolded proteins that do not refold, or are unsuccessfully refolded are targeted for proteolytic degradation^3–5^ or may form aggregates. In model eukaryotic cells such as budding yeast, these aggregates have distinct cellular localization, and in bacillary bacteria, aggregates, known as ‘inclusion bodies’, are identified at the poles^1^. There is ongoing debate regarding the physiological role, if any, of protein aggregates. Asymmetric inheritance of aggregates results in hallmarks of aging and fitness defects in the cell accumulating the aggregates, and rejuvenation in its sister^6,7^. However, although oxidised proteins may be irreversibly damaged^1,7^, aggregates formed, for example following heat shock, are capable of refolding at a later time^8^, suggesting that different types of aggregate may have both differing fates and consequences on cell viability and physiology.

Compared with the clear role of HSPs in protein refolding, the role of small heat shock proteins (sHSPs) in proteostasis is less evident^9^. Small HSPs act in an ATP-independent manner, and have been associated with the formation of cellular protein aggregates in both bacterial and eukaryotic model systems^10,11^. Biochemical studies performed *in vitro* suggested that sHSPs bind unfolded proteins, keep them in a near-native state, and thereby facilitate their refolding by disaggregating ATP-dependent HSP chaperones^9,12^. Recent work in yeast has suggested that the sHSP Hsp42 acts as an aggregase. There are distinct forms of aggregates in yeast, with defined locations. Hsp42 was necessary for both the formation and trafficking of one type of aggregate, termed CytoQs^13,14^, and its pro-aggregase function could be recapitulated *in vitro* using a model substrate^15^. However, whether other sHSPs also perform pro-aggregase functions is not known.

Here, we investigated the role of HspX, a *Mycobacterium smegmatis* small heat shock protein in the formation and trafficking of protein aggregates. HspX was required for the polar localization of both a model aggregation-prone protein, and several native mycobacterial protein aggregates. Furthermore, we performed semi-quantitative mass-spectrometry of *M. smegmatis* protein aggregates. We identified a subset of aggregates that appeared to be HspX-dependent, suggesting that HspX acts both as a sortase and/or pro-aggregase for a subset of mycobacterial protein substrates.

## Results

### HspX is necessary for the polar localization of an aggregation-prone protein in *M. smegmatis*

A number of fluorescent reporters have been used to study protein aggregation in bacterial and eukaryotic systems. Many, such as the widely used firefly luciferase^16^ require physiological stress such as heat shock to promote aggregation of the reporter^9^, although mCerulean fused to ELK16 could aggregate without heatshock^16^. To construct a new reporter that was aggregation-prone in the absence of heat shock, we mutated a number of conserved residues in green fluorescent protein (GFP). Mutation of the highly conserved aspartate at residue 103 to asparagine resulted in both loss of green fluorescence by two orders of magnitude, and aggregation of the protein when expressed in wild-type *Mycobacterium smegmatis*. Since the resulting GFP-D103N was no longer fluorescent, we C-terminally fused the protein with a flexible linker and monomeric red fluorescent protein (mRFP), so as to be able to visualize the expressed product, which we named GLR103 (green-linker-red, 103^rd^ residue mutated), and its corresponding wild-type dual-fluorescent reporter as GLR. We confirmed that when expressed in *M. smegmatis*, a large proportion of GLR103 formed insoluble aggregates compared with GLR, as determined by differential ultra-centrifugation (Fig. S1a). Measurement of red fluorescence in the different sub-cellular fractions confirmed that GLR103 within the insoluble fraction, which would contain large aggregates, contained the major fluorescent fraction in wild-type cells (Fig. S1b), and therefore the reporter would be suitable for tracking aggregate formation by microscopy.

**Figure 1:**
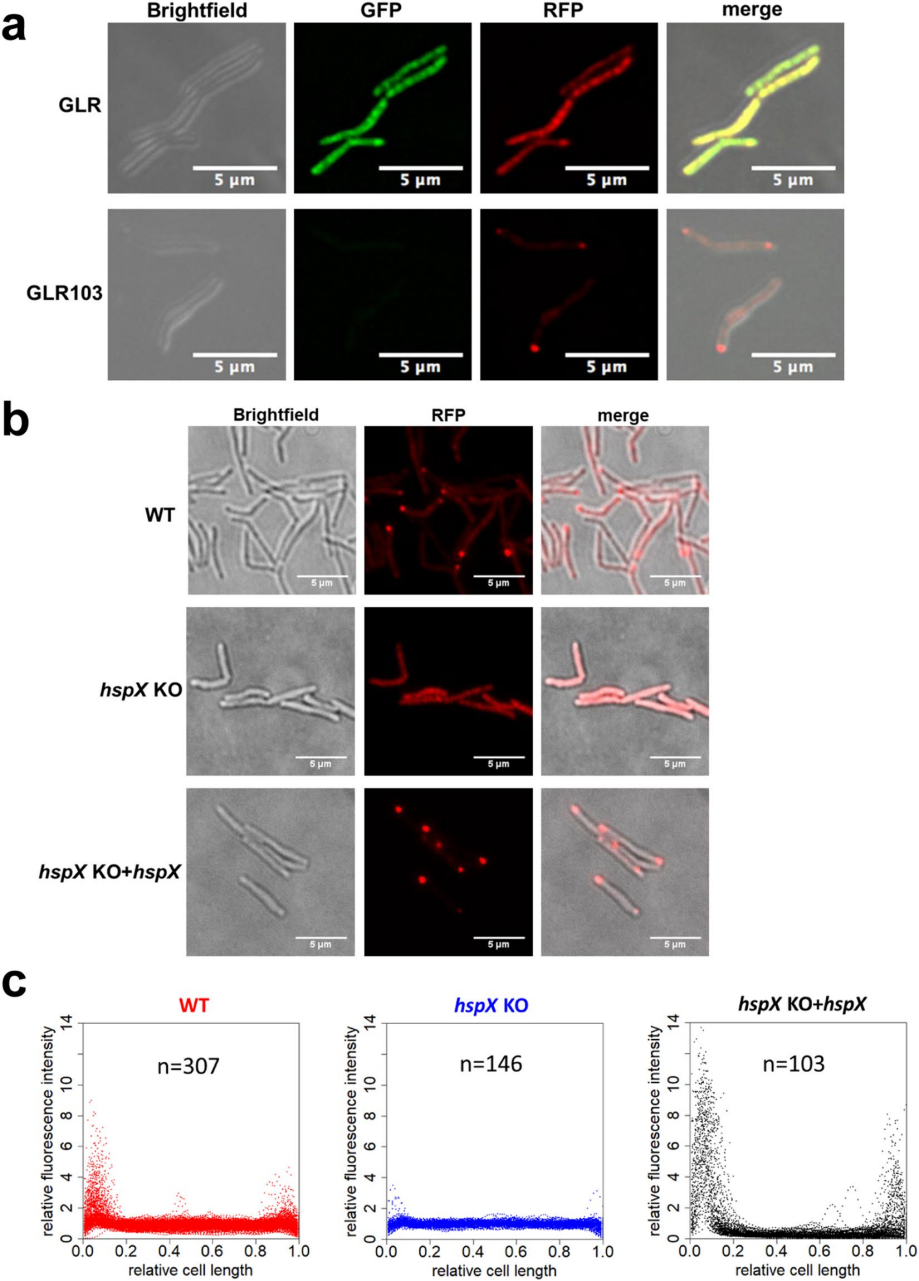
HspX promotes polar localization of protein aggregates. (**a**) Fluorescence microscopy images of wild-type *M. smegmatis* expressing dual fluorescent GLR (top panel) or mutated GLR103 proteins. Images illustrative of >300 individual cells. (**b**) Fluorescence microscopy of wild-type *M. smegmatis* (WT), *hspX*-deleted *M. smegmatis* (*hspX* KO) and complemented (*hspX* KO + *hspX*) strains expressing GLR103. Images illustrative of >100 cells. (**c**) Analysis of red channel fluorescence intensity from GLR103 across the normalized cell length of images represented in (**b**) of the three strains. The y-axis represents relative red fluorescence along the cell axis compared with the mean fluorescence intensity of the cell being analyzed (see Methods). The symbol (n) represents number of individual cells analyzed.

Expression of wild-type GLR in wild-type *M. smegmatis* resulted in distribution of the dual-fluorescent protein throughout the cell (Fig. 1a). By contrast, expression of GLR103 resulted in discrete foci of red fluorescence at the pole (Fig. 1a), in keeping with the known polar distribution of protein aggregates in bacteria including mycobacteria^6,7,16,17^. To determine whether the sHSP HspX was involved in aggregation of GLR103, we constructed a strain of *M. smegmatis* in which *hspX* was deleted, ∆*hspX*. Deletion of *hspX* did not cause a growth defect when the strains were grown under standard laboratory medium and conditions (Fig. S2). Unlike in wild-type *M. smegmatis*, expression of GLR103 in ∆*hspX* did not result in polar distribution of the fluorescent protein (Fig. 1b,c). When expressed in ∆*hspX*, GLR103 was less prone to aggregation (Fig. S1). Complementation of the deletion strain with *hspX* rescued the localization phenotype, confirming a role for HspX in the polar localization of large protein aggregates (Fig. 1b,c).

### The N-terminus of mycobacterial HspX is necessary but not sufficient for its polar localization

**Figure 2:**
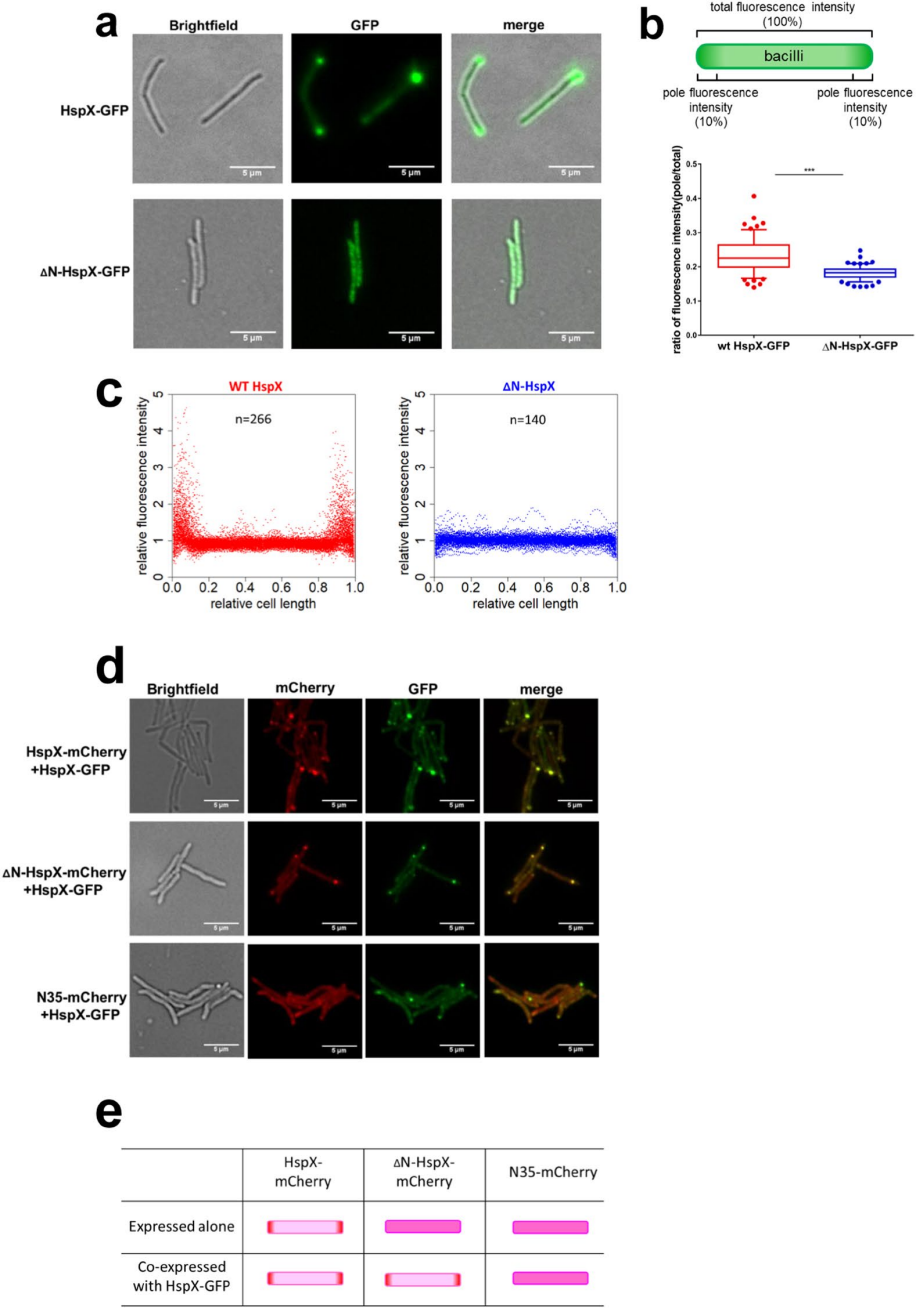
The N-terminal domain of HspX is necessary but not sufficient for its polar localization. (**a**) Fluorescence microscopy of *M. smegmatis*-*∆hspX* expressing either full-length HspX-GFP (HspX-GFP) or HspX-GFP missing the N-terminal 35 amino acids (∆N35-HspX-GFP). Images representative of >140 cells analyzed. (**b**) Cartoon representing analysis of relative polar vs. non-polar localization of green fluorescence in cells imaged in (**a**) – upper panel. Box and whisker plot of images analyzed according to the schematic (lower panel). Box represents inter-quartile range, with line representing median, and whiskers represent 95% confidence intervals. ***p < 0.001 by two-tailed Student’s t-test. (**c**) Analysis of green channel fluorescence intensity from HspX-GFP or ∆N35-HspX-GFP across the normalized cell length of images represented in (**a**), the symbol (n) represents number of individual cells analyzed. (**d**) Fluorescence microscopy of *M. smegmatis*-*∆hspX* expressing combinations of mCherry or GFP tagged full-length or truncated constructs of HspX as per the legend. (**e**) Cartoon schematic of model representing polar localization of oligomeric HspX. Full-length HspX can rescue the polar localization defect of ∆N35-HspX, presumably via formation of hetero-oligomers that are competent for trafficking to the pole.

HspX has three domains, a conserved α-crystallin domain, flanked by N- and C- terminal domains^18–21^. The N-terminus of the *M. tuberculosis* homolog of HspX was reported to play a role in its chaperone activity, as well as self-oligomerization^20,22^. Furthermore, the N-terminus of a member of the yeast sHSP family, Hsp42, played a crucial role in peripheral aggregate formation^14,23^, and the N-terminal domains of other sHSPs have been reported to mediate substrate interaction and self-assembly^18,19,21^. We therefore decided to test the localization of C-terminally GFP-tagged HspX, with the N-terminal 35 residues deleted (∆N35-HspX-GFP), compared with the full-length protein (HspX-GFP), both expressed on an otherwise HspX-null background. Full-length HspX-GFP localized to the bacterial pole, and was functional since it complemented the *hspX*-null strain (Fig. S3a). However, deletion of the N-terminus abrogated polar localization of HspX-GFP (Fig. 2a–c), or of GLR103 (Fig. S3b). This was not due to lower expression levels of the N35 deletion mutant (Fig. S4). Since sHSPs are known to assemble into homo-oligomers^20–22,24^, we wished to determine if self-assembly was associated with localization. We C-terminally tagged full-length HspX, ∆N35-HspX, and just the 35 N-terminal residues of HspX (N35) with monomericCherry fluorescent protein (mCherry). We then expressed these constructs +/− full-length HspX-GFP, on an otherwise ∆*hspX* background. As before with the GFP-tagged constructs, HspX-mCherry, but not ∆N35-HspX-mCherry localized to the bacterial poles, and only full-length HspX-mCherry was found in the insoluble fraction of the cells. The 35 N-terminal residues of HspX were not sufficient for polar localization of N35-mCherry, suggesting that other molecular determinants in the full-length HspX protein were also necessary for its localization (Fig. S5). However, when co-expressed with full-length HspX-GFP, both HspX-mCherry and ∆N35-HspX-mCherry co-localized to the pole (Fig. 2d). These data suggested that full-length and N-terminally deleted HspX were able to co-assemble, and the hetero-oligomers were competent for polar localization (Fig. 2e).

### HspX has a functional role in the localization of large mycobacterial protein aggregates

**Figure 3:**
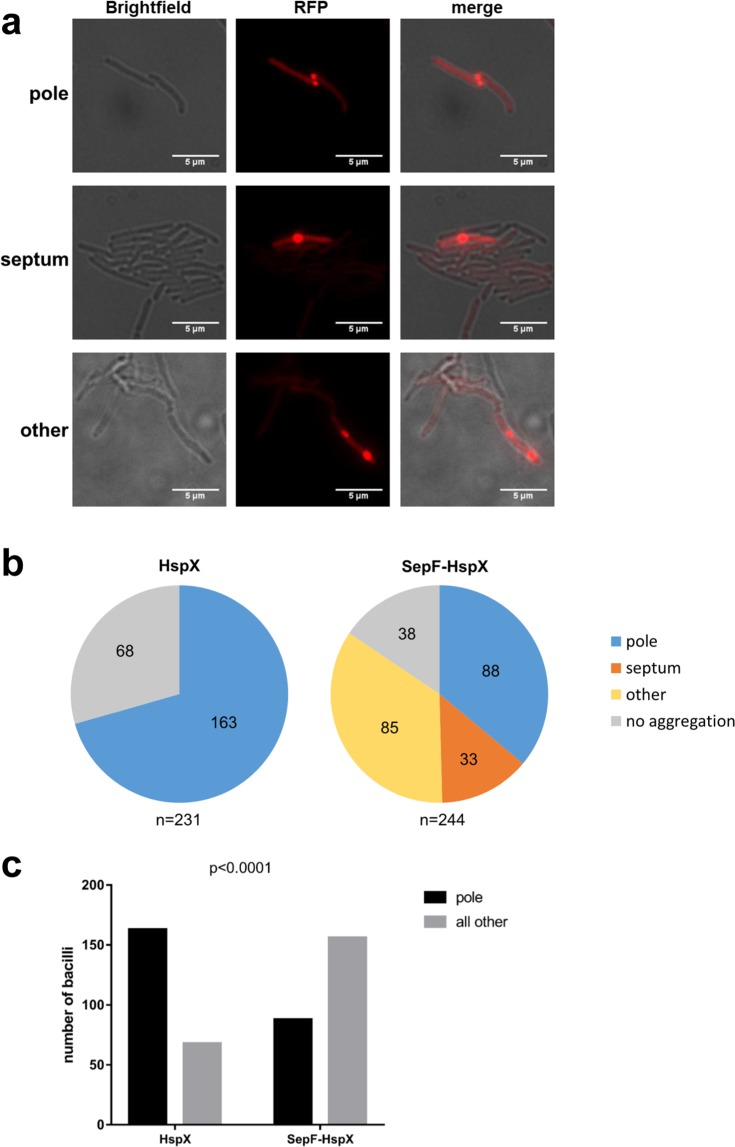
HspX has a functional role in the polar localization of protein aggregates. (**a**) Fluorescence microscopy of *M. smegmatis*-*∆hspX* expressing SepF-HspX and GLR103. Images representative of polar or septal or “other” localization of GLR103 aggregates are shown. (**b**) Pie-charts showing relative distribution of aggregates of GLR103 expressed in *M. smegmatis*-*∆hspX* and complemented either with wild-type HspX or SepF-HspX. The “other” designation was for cells with clearly visible aggregates that were not solely at the pole or septum – see (**a**) for representative example. The symbol (n) represents number of individual cells analyzed. (**c**) Bar chart comparing polar vs “all other” (including septal and no aggregation) distribution of aggregates of cells analyzed in (**b**) and tested for statistical significance (p < 0.0001) by Fischer’s exact test.

We wished to determine whether HspX played a causal role in polar aggregate formation. Given the generally ascribed chaperone role for sHSPs, one possibility was that HspX associates with misfolded proteins in a non-causal, but consequential role, similarly to the sHSPs IbpA and IbpB in *E. coli*^6^. Alternatively, HspX may play a directly causal role in the sorting of misfolded protein aggregates to the pole. To resolve these two hypotheses, we decided to deliver HspX to an alternate cellular location by fusing it with SepF, a protein known to localize to the mycobacterial septum^25^. SepF-GFP localized to the septum (Fig. S6a), but did not alter localization of GLR103 (Fig. S6b). SepF-HspX-GFP showed very little expression, for reasons that were not clear, therefore we fused SepF to HspX alone. When co-expressed with GLR103 on an *hspX* null background, SepF-HspX significantly altered the localization of the GLR103 aggregates to multiple cellular locations, including to the cellular septum (Fig. 3a,b), and aggregates were significantly less associated with the poles (Fig. 3c). Taken together, these data support a critical role for mycobacterial HspX in the sorting and distribution of model protein aggregates to the bacterial pole.

### HspX expression promotes the aggregation and polar sorting of a subset of cellular proteins

To investigate whether HspX played a role in the formation of aggregates of native mycobacterial proteins, we isolated the insoluble protein fraction, representing aggregates, in wild-type *M. smegmatis*, and in strains deleted for, and over-expressing *hspX* (Fig. S7) and subjected them to semi-quantitative mass-spectrometry by tandem mass-tags. In total, the insoluble fraction contained 1355 proteins with >1 unique peptides that were identified in all three conditions (Table S1). Only a subset of these proteins varied quantitatively by *hspX* expression. The vast majority of proteins that varied were over-represented in *hspX* over-expression, or wild-type *M. smegmatis* compared with ∆*hspX*. Of these, 251 varied by over 1.5-fold in the *hspX* over-expression strain (HspX-OE) compared with ∆*hspX* (Table S2). Of note, most proteins that were over-represented in HspX-OE were also over-represented in wild-type *M. smegmatis* compared with ∆*hspX*, but at a smaller differential ratio (Table S2), suggesting a causal role for HspX in the generation of aggregates in this subset of proteins.

**Figure 4:**
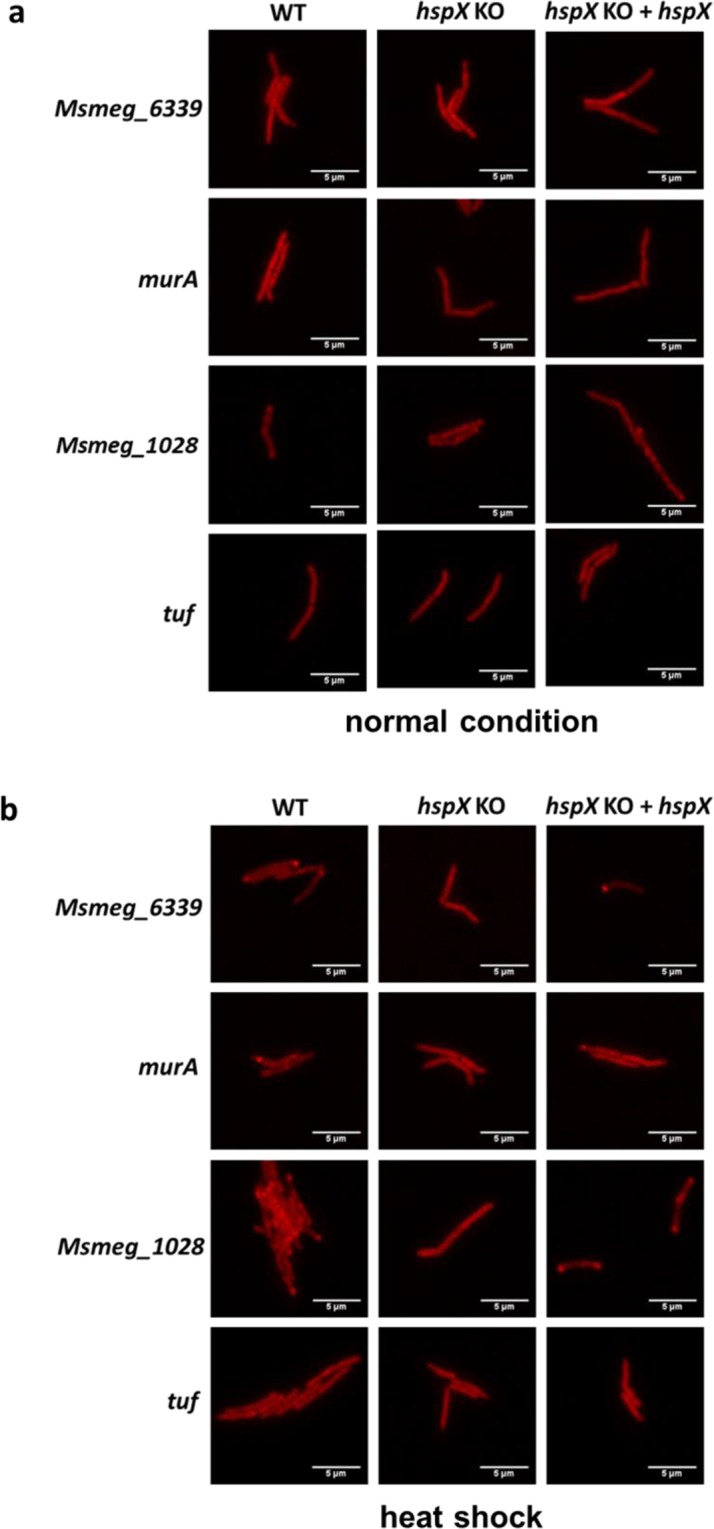
HspX acts as a polar sortase for native-protein aggregates. Representative fluorescence microscopy images of wild-type (WT) *M. smegmatis*, *hspX*-deleted *M. smegmatis* (*hspX* KO) and complemented (*hspX* KO + *hspX*) strains expressing C-terminal mApple-tagged proteins (*Msmeg_6339, murA, tuf* and *Msmeg_1028*) following axenic growth at 37 °C (**a**) or after 1 hr heat-shock pulse at 45 °C (**b**). See Fig. S8 for quantification.

For the model protein GLR103, HspX sorted GLR103 aggregates to the mycobacterial poles. Did HspX perform a similar function in any native proteins? We tested 4 candidates that were identified in the mass spectrometry analysis as present in the insoluble fraction by C-terminally fusing them with fluorescent mApple and visualizing by microscopy (Figs 4 and S8a). When grown under standard laboratory conditions, none of the four proteins formed large, visible aggregates (Fig. 4a). However, following a heat-shock pulse, three of the four candidates, with the exception of EF-Tu (coded by *tuf*) formed large visible aggregates that were localized at the cellular pole in an HspX-dependent manner (Figs 4b and S8b). Therefore HspX also acts as a polar sortase for native, aggregation-prone mycobacterial proteins.

## Discussion

Protein misfolding may occur due to environmental stressors, such as oxidation or heat shock, or due to translational errors from antibiotics, mutations in the translation apparatus, or stochastic mistakes^1,7,26–30^. The polar localization of protein aggregates in bacteria, including mycobacteria is a long-observed phenomenon, but the molecular mechanisms for sorting misfolded proteins to the poles is less clearly understood. In *E. coli*, the sHSPs IbpA and IbpB were associated with protein aggregate formation^6,10^, and co-localized with inclusion bodies at the cellular poles. However, the sorting of aggregates in *E. coli* to the poles appeared to be a passive process, driven predominantly by nucleoid occlusion^17^. In mycobacteria, depletion of the HSP70 DnaK led to a marked increase in aggregate formation, consistent with its known critical role as a disaggregase^16^. This was associated with upregulation and redistribution of the Hsp100 homologue ClpB^31^ to discrete foci^16^. ClpB was also shown to associate with irreversibly carbonylated proteins that result from oxidation^7^. However, expression of an enzymatically null ClpB did not associate with aggregates, nor was functional ClpB required for aggregate formation or polar localization^7^, suggesting that ClpB, as a refoldase, was necessary for dealing with aggregate-induced stress, but not the formation or sorting of aggregates to the mycobacterial pole. Similarly to Hsp42 in yeast^13,14^, mycobacterial HspX is required for the polar localization of both a model aggregation-prone protein, GLR103 and native protein aggregates (Figs 1 and 4). Redistribution of HspX results in mis-sorting of GLR103, verifying its active role in sorting this model protein (Fig. 3). Deletion of HspX led a much smaller fraction of GLR103 being found in the insoluble fraction. Therefore, in addition to its function as a sortase for this model protein, it seems likely that HspX acted as a pro-aggregase in this context.

The subdomains of sHSPs, including HspX have been shown to be critically important for their functions. Both N- and C-terminal domains of HspX have been shown to contribute to oligomerization and chaperone functions *in vitro*^20–22,24,32^. The N-terminus of yeast Hsp42 contains both a prion-like domain and an intrinsically disordered domain, which contribute to CytoQ aggregate formation and localization^23^. By expressing differentially-tagged HspX and truncation proteins in *M. smegmatis* cells lacking endogenous HspX, we showed that N-terminal domain of HspX was necessary, but not sufficient for its polar localization. However, HspX lacking the N-terminal domain could co-localize to the pole when co-expressed with wild-type HspX – suggesting that it is the oligomeric form of the protein that is located at the poles, and that the N-terminally truncated protein can form hetero-oligomers with full-length HspX (Fig. 2). Whether oligomeric HspX is sufficient for polar localization, or requires binding to other partners to sort to the pole is not known.

We performed semi-quantitative mass-spectrometry of *M. smegmatis* aggregates. A prior study on the role of DnaK in mycobacterial aggregate formation showed that depletion of DnaK appeared to cause large, global changes in mycobacterial aggregates, although formal quantitation was not performed^16^. By contrast, deletion or over-expression of HspX did not quantitatively change the majority of detected insoluble proteins (Table S1). This is perhaps not surprising for two reasons. The experiment was performed in the absence of proteotoxic stress. Also, the function of DnaK as a disaggregase will likely have a greater impact on the quantity and number of aggregated proteins compared with HspX in the absence of proteotoxic stress.

We chose to perform the experiment in the absence of proteotoxic stress to identify “aggregation-prone” client proteins of HspX. Some cellular proteins were under-represented in aggregates when *hspX* was deleted and were over-represented when *hspX* was over-expressed (Table S2), suggesting that at least for this subset of proteins, HspX may act as an aggregase. However, as already noted the experiment was performed under standard laboratory growth conditions, and it is possible that with greater proteotoxic stress, the number of HspX client proteins may be increased. We tagged a number of proteins that we identified as present in *M. smegmatis* aggregates with a fluorescent protein to allow visualization. Under standard laboratory growth conditions, no aggregates were visualized by microscopy: suggesting that the majority of tagged protein molecules had folded appropriately and were soluble, although we could not exclude small aggregates that were not visually distinguishable trafficking to the pole. However, under proteotoxic stress (heat shock pulse), three of the four proteins tested formed large visible aggregates, suggesting that our dataset probably contains “aggregation-prone” proteins, and formation and/or sorting of these aggregates to the mycobacterial poles was HspX-dependent, confirming its role as an aggregate polar sortase for native mycobacterial proteins.

HspX has been intensively studied. It is expressed abundantly under clinically relevant environmental stressors such as hypoxia due its regulation by the DosRST system^33–36^, and some tuberculosis vaccine candidates include HspX as an immunogen^37^. The cellular function of HspX, however, has not been characterized to date. Deletion of aggregation-associated proteins lead to fitness defects under stress in a number of model systems. In yeast, deletion of Hsp42 led to a specific fitness defect upon cyclic heat shock, but not when strains were grown at 30 °C^15^, and this was dependent on the protein’s N-terminal disordered domains^23^. Similarly, ClpB depletion in *M. tuberculosis* resulted in a defect in recovery from late stationary phase and in mouse lungs^7^. Deletion or chemical inhibition *in vitro* of HspX in *M. tuberculosis* decreased viability in hypoxia^36,38^. When mice were infected by an HspX-deficient strain of *M. tuberculosis*, the strain grew to a greater organ burden compared with the wild-type parent strain, but was more rapidly cleared upon antibiotic treatment, suggesting that HspX played potentially opposing roles in fitness to the host versus antibiotics in that model^39^. By sorting insoluble protein aggregates to a distinct cellular location, HspX acts to compartmentalize the cell in the absence of membrane-bound organelles. As such, HspX may be a key mycobacterial driver of phase separation^40,41^, which has been associated with potentially adaptive functions of aggregate formation^42^.

## Materials and Methods

### Bacterial strains, culture conditions, plasmids and growth measurement

*Mycobacterium smegmatis* mc^2^-155 (ATCC) was grown in Middlebrook 7H9 liquid broth supplemented with: 10% ADS (albumin-dextrose-salt), 0.2% glycerol and 0.05% Tween-80, or plated on LB (Lennox) agar. *E. coli* DH5α (CW Biotech) was used for cloning. Antibiotic concentrations for *M. smegmatis* were: 50 μg ml^−1^ hygromycin, 20 μg ml^−1^ streptomycin, 25 μg ml^−1^ kanamycin, zeocin 50 μg ml^−1^. Antibiotic concentrations for *E. coli* were: 150 μg ml^−1^ hygromycin, 50 μg ml^−1^ streptomycin, 50 μg ml^−1^ kanamycin, zeocin 50 μg ml^−1^. All strains were grown at 37 °C unless otherwise indicated. For the induction of the tetracycline-inducible promoter, after growth overnight at 37 °C, *M. smegmatis* strains were subcultured in fresh media and anhydrotetracycline – ATc (50 ng/mL) was added to the media 3 hours prior to further experimental manipulation. For the heat pulse experiments (Fig. 4), cultures were shifted to 45 °C with shaking for one hour, and then immediately used for microscopy (see below).

All genes were cloned by PCR from *M. smegmatis* genomic DNA or a template plasmid and verified by Sanger sequencing (see Table). The fluorescent protein reporter GLR was constructed by fusion PCR and GLR103 was constructed by site-directed-mutation PCR. HspX-GFP was made by fusing GFP to the C-terminal of HspX by PCR. DeltaN-HspX-GFP was made by deleting the first 35 amino acids of HspX based on HspX-GFP by PCR. HspX-mCherry was made by fusing mCherry to the C-terminal of HspX by PCR. DeltaN-HspX-mCherry was made by deleting the first 35 amino acids of HspX based on HspX-mCherry by PCR. N35-HspX-mCherry was made by fusing mCherry to the N-terminal first 35 amino acids of HspX by PCR. SepF fragment was got by PCR using mc^2^155 strain genomic DNA as template. SepF-HspX was made by fusing HspX to the C-terminus of SepF. Initially, a construct, SepF-HspX-GFP was made, but this failed to localize to the septum for unknown reasons, and therefore the SepF-HspX construct was tested. The mApple fragment was obtained by PCR (with start codon deleted) using pMV261-mApple (a kind gift from Eric J. Rubin) as template and then inserted into plasmid pSML1357 by enzyme digestion (NheI and HindIII) and ligation. *Msmeg_6339*, *murA* and *tuf* (with stop codons deleted) were obtained by PCR using *M. smegmatis* genomic DNA as template and then inserted into plasmid pSML1357 by enzyme digestion (PacI and NheI) immediately 5′ to *mApple* to make a fusion protein of Msmeg_6339/MurA/EF-Tu-mApple. Plasmids were transformed *M. smegmatis* competent cells by electroporation using standard methodology. Growth of bacteria was measured by measuring the optical density of bacteria media at 600 nm wavelength. Plasmids and primers are listed in Tables 1 and 2 respectively.

**Table 1:** Plasmids used in this study.

Name	Description	Source
pUVtetOR	Episomal mycobacterial expression vector with tet^ON^ inducible system, Hyg^R^	Ref.^2^
pUVtetOR-GFP-linker-RFP (GFP-mRFP)	pUVtetOR containing GFP translationally fused with RFP through a long flexible linker (GGSGGGGSGGGSSGG), Hyg^R^	This Study
pUVtetOR-GLR-D103N (GFP-D103N-mRFP – also called GLR103)	Mutation of D103N on GFP of pUVtetOR-GFP-linker-RFP, Hyg^R^	This Study
pUVtetOR-GLR103-HspX	Vector expressing both HspX and GLR103 reporter, Hyg^R^	This Study
pUVtetOR-HspX-GFP	Vector expressing HspX C-terminal fused with GFP, Hyg^R^	This Study
pUVtetOR-ΔN HspX-GFP	Vector expressing N-terminal truncated HspX C-terminal fused with GFP, Hyg^R^	This Study
pUVtetOR-ΔC HspX-GFP	Vector expressing C-terminal truncated HspX C-terminal fused with GFP, Hyg^R^	This Study
pUVtetOR-ΔNΔC HspX-GFP	Vector expressing N-terminal, C-terminal truncated HspX C-terminal fused with GFP, Hyg^R^	This Study
pML1357	Integrative mycobacterial expression vector, Hyg^R^	Addgene
pSML1357	Integrative mycobacterial expression vector with selection marker changed from original Hyg^R^ to Strep^R^	This Study
pSML1357-HspX-mcherry	Vector expressing HspX C-terminal fused with mcherry, Strep^R^	This Study
pSML1357-ΔN HspX-mcherry	Vector expressing N-terminal truncated HspX C-terminal fused with mcherry, Strep^R^	This Study
pSML1357-N35 HspX-mcherry	Vector expressing N-terminal HspX C-terminal fused with mcherry, Strep^R^	This Study
pUVtetOR-GLR103-SepF-linker-HspX	Vector with ATC induced expression of GLR103 reporter and SepF-linker-HspX fusion construct. Hyg^R^	This Study
pSML1357-Msmeg_6339-mApple	Vector expressing mApple C-terminal fused with Msmeg_6339, Strep^R^	This Study
pSML1357-murA-mApple	Vector expressing mApple C-terminal fused with murA, Strep^R^	This Study
pSML1357-tuf-mApple	Vector expressing mApple C-terminal fused with tuf, Strep^R^	This Study
pSML1357- Msmeg_1028-mApple	Vector expressing mApple C-terminal fused with Msmeg_1028, Strep^R^

**Table 2:** Primers used in this study.

Name	Sequence 5′-3′
GFP-PacI-F	AATGTTAATTAAGAAGGAGATATACATCATGTCGAAGGGCGAGGAGCTGTTC
GFP-linker-R	GAGCCGCCGCCCGAGCCGCCGCCGCCCGAGCCGCCCTTGTACAGCTCGTCCATGCC
mRFP-linker-F	GCGGCGGCTCGGGCGGCGGCTCGTCGGGCGGCGCCTCCTCCGAGGACGTCATCAAG
mRFP-EcoRV-R	AATCTTGATATCTTAGCGCCGGTGGAGTGGCGG
GFP-D103N-Mut-F	CCTTCAAGGACAACGGTAACTACAAGAC
GFP-D103N-Mut-R	GTCTTGTAGTTACCGTTGTCCTTGAAGG
BamHI-HspX-F	AACACGGATCCAATGACCAAACTTCCTGAAC
HspX-GFP-R	GGTGGCCACCGGCGGGTCCGGGCTGACGGTCTCCACCGCG
HspX-GFP-F	GACCCGCCGGTGGCCACCATGTCGAAGGGCGAGGAGC
HindIII-GFP-R	TTCGCTAAGCTTCTACTTGTACAGCTCGTCC
HspXKO-1	ACCGTGCGGCACGGGGAGAT
HspXKO-2	GGCCTCTCGAAGCGGTCCTCCTCACTCGTAGG
HspXKO-3	AGGAGGACCGCTTCGAGAGGCCTATAACTTCG
HspXKO-4	CGGCCATGGCGAAGTACTTCTAGACTCGAGATAACTTCG
HspXKO-5	TCTAGAAGTACTTCGCCATGGCCGTGAACA
HspXKO-6	GCCCGGTGCCCATGGAC
RBS-HspX-GFP-F	AAGGAGATATACATCATGACCAAACTTCCTGAACGATCACGAG
pUVtet-△N35HspX-GFPa	ATAGGCTCTGGGAGTACCCG GACCACATCATCCGGATCGAGG
pUVtet-△N35HspX-GFPb	GAAAAGTTCTTCTCCTTTACT CGGGCTGACGGTCTCCACCGCG
pUVtet-△N35HspX-GFPc	CGGTGGAGACCGTCAGCCCG AGTAAAGGAGAAGAACTTTTCAC
pUVtet-△N35HspX-GFPd	CCCCAATTAATTAGCTAAA TCAACATTTGTATAGTTCATC
pUVtet-HspX-GFPe	CGGGTACTCCCAGAGCCTATCTATC
pUVtet-HspX-GFPf	TTTAGCTAATTAATTGGGGACCC
pUVtet-△C HspX-GFPa	ATAGGCTCTGGGAGTACCCG ATGACCAAACTTCCTGAACGATC
pUVtet-△C HspX-GFPb	GAAAAGTTCTTCTCCTTTACT GAACCCGAGGCAGCCGCCGAGAAG
pUVtet-△C HspX-GFPc	CCCGAGGCAGCCGCCGAGAA AGTAAAGGAGAAGAACTTTTCAC
pUVtet-△C HspX-GFPd	CCCCAATTAATTAGCTAAA TCAACATTTGTATAGTTCATC
pUVtet-△N△CHspX-GFPa	ATAGGCTCTGGGAGTACCCG GACCACATCATCCGGATCGAGG
pUVtet-△N△CHspX-GFPb	GAAAAGTTCTTCTCCTTTACT GAACCCGAGGCAGCCGCCGAGAAG
pUVtet-△N△CHspX-GFPc	CCCGAGGCAGCCGCCGAGAA AGTAAAGGAGAAGAACTTTTCAC
pUVtet-△N△CHspX-GFPd	CCCCAATTAATTAGCTAAA TCAACATTTGTATAGTTCATC
mcherry-wtHspX-R	GACGTCCTCGGAGGAGGCCGGGCTGACGGTCTCCACCGCGACA
wtHspX-mcherry-F	GTGGAGACCGTCAGCCCGGCCTCCTCCGAGGACGTCATCAAGG
HindIII-mcherry-R	CCCAAGCTT TTA ACTGGATCCGCTAGATCCCTGGGAGCC
mcherry-N35HspX-R	GAC GTC CTC GGA GGA GGC ACC GAA AAC CGG GCG GAT CGA GGC C
N35HspX-mcherry-F	ATC CGC CCG GTT TTC GGT GCC TCC TCC GAG GAC GTC ATC AAG G
PacI-RBS-△NHspX-F	GCC TTA ATT AAG AAG GAG ATA TAC ATC GAC CAC ATC ATC CGG ATC GAG GAC GA
PacI-SepF-F	CCTTAATTAACAGAAAGGAGGTTAATAATGAGCACACTGCATAAGGTCAAGGCC
SepF-linker-HspX-R	TTGGTGCCGCCCGACGAGCCGCCACGGTAGGAGTAGAAGCCCGCCTCGG
Linker-HspX-F	GGCGGCTCGTCGGGCGGCACCAAACTTCCTGAACGATCACGAGCACGC
HspX-HindIII-R	CCCAAGCTTTTACGGGCTGACGGTCTCCA
GLR103-SepF-F	ACCGGCGCCTAAGATCAGAAAGGAGGTTAATAATGAGCAC
HspX-Ptet R	GGTCCCCAATTAATTAGCTAAAGCTTGATTTACGGGCTGACGGTCTC
mApple-F	CTA GCT AGC AGC AAG GGC GAG GAG AAT AAC ATG GCC ATC ATC AAG GAG TTC ATG CGC
mApple-R	CCC AAG CTT TTA GGT CGA GTC CAG GCC CAG CAG CGG GTT CGG GAT CGG CTT GCC CTT GTA CAG
Msmeg_6339-F	CCTTAATTAAGAAGGAGATATACATCatggagtcgaacccaccgagtgccgtcgtcga
Msmeg_6339-R	CTAGCTAGCgggcgtgacgatgctgaagttcgggt
murA-F	CCTTAATTAAGAAGGAGATATACATCgtgagcgagcgtttcgtggtgaccggtggcaa
murA-R	CTAGCTAGCcgagcttactctctcgatctcggctc
tuf-F	CCTTAATTAAGAAGGAGATATACATCgtggcgaaggcgaagttcgagcggacgaagcc
tuf-R	CTAGCTAGCcttgatgatcttggtgacgcggccgg

### *hspX* deletion strain construction

The *M. smegmatis* gene *hspX* (*MSMEG_3932*) was deleted through linear dsDNA guided homologous recombineering^30,43,44^. Briefly, a Zeocin resistant cassette flanking the 500 bp upstream and downstream region of *MSMEG_3932* was used as an allele exchange substrate (AES). The upstream 500 bp and downstream 500 bp of *MSMEG_3932* were amplified from boiled mc^2^155 strain by PCR. The Zeocin resistant cassette was amplified from a plasmid pKM-Zeo-lox (A kind gift from Eric J. Rubin). The three fragments were fused together by overlap-PCR and verified by sequencing. *M.smegmatis* mc^2^155 transformed with the recombinase expressing plasmid pNIT(KanR)::recET::sacBR was induced to express RecET recombinase overnight with 10 nM Isovaleronitrile (IVN), from which competent cells were prepared and then electroporated with the KO cassette. The transformants were allowed to recover in 7H9 for 4 hours and then grow on LB-agar plates supplemented with 25 μg/mL Zeocin. The strain construction was confirmed by PCR, which verified both integration of the cassette and that it was in the appropriate genomic context. The HspX deleted strain was cured of the recombinase expressing plasmid before downstream applications.

### Western blot analysis

Mycobacteria were lysed by beads beating in 25 mM Tris-HCl, 100 mM NaCl and 1 mM EDTA with protease inhibitor (Roche). Lysates for Fig. S4 were quantified by Bradford assay kit (Bio-Rad), loaded in SDS-PAGE gel and immunoblotted with HRP-conjugated anti-GFP antibody (Clone LGB-1, CWbio) or HRP-conjugated anti-rpoB antibody (clone 8RB13, Santa CruZ Biotechnology). Blots were visualized using ECL reagents (CWbio).

### Subcellular fractionation

Methods were adopted from^16^ with a few modifications. Briefly, mycobacterial cultures of interest were pelleted at 4 °C and washed with fractionation buffer (50 mM Tris, 100 mM NaCl, 10% glycerol, 2 mM EDTA, pH 8, supplemented with protease inhibitor cocktails (Roche)). Cell pellets were re-suspended in 1 mL fractionation buffer and subjected to four times of 40 seconds beads beating (MiniBeadbeater-16, 230 V. BioSpec) with 90 seconds intervals on ice in between. Beads and non-lysed cells were discarded by centrifugation at 3,000 × g for 5 minutes at 4 °C, the supernatant was collected and another round of low speed (5,000 × g) centrifugation for 20 minutes at 4 °C was used to remove the residual debris. Supernatants were collected and transferred to 1.5 mL Beckman tubes, and then centrifuged at 180,000Xg for 2.5 hours at 4 °C. Supernatants from this step were collected, labeled as “Soluble” fraction and kept at −20C. Pellets were carefully resuspended in 0.5 mL ice-cold fractionation buffer supplemented with 1% TritonX-100, allowed to solubilize at 4 °C with gentle shaking for 1 hour. The whole suspension was then centrifuged at 20,000 × g for 2 hours at 4 °C. The supernatant of this fractionation was collected and labeled as “Membrane” fraction, the pellets were dissolved in ice-cold fractionation buffer supplemented with 1% SDS, and labeled as “Pelleted” fraction. All three fractions of different bacterial cultures were loaded onto an SDS-PAGE gel for electrophoresis. The GFP-mRFP (or mutant) proteins were visualized by western blotting using standard protocols and mouse mono-clonal anti-GFP antibody as above.

### Quantitative mass-spectrometry

Two independent cultures of each of wild-type *M. smegmatis, hspX* knock-out and *hspX* over-expression strains were grown in complete 7H9 medium to late log phase and then lysed and processed as detailed above for “sub-cellular fractionation”. The “insoluble” fractions were resuspended and solubilized using 8 M urea, quantified using Bradford assay and then equal quantities loaded onto an SDS gel and subjected to PAGE. Following SDS-PAGE, all the lanes were excised into bands, reduced with 25 mM dithiotreitol and alkylated with 55 mM iodoacetamide. In-gel digestion was then performed using sequencing grade modified trypsin in 50 mM ammonium carbonate buffer at 37 °C overnight. Peptides were extracted twice with 0.1% trifluoroacetic acid in 50% acetonitrile aqueous solution for 30 minutes. Extracts were then partially dried in a speedivac to reduce volume. Tryptic peptides were then redissolved in 50 µl 200 mM tetraethylammonium bromide (TEAB) and then 2 µl TMTsixplex reagent was added to each sample according to the manufacturer’s instructions. The reaction mixture was incubated at room temperature for 1 hour, following which 0.5 µl of 5% hydroxylamine (pH 9) was added to the reaction mixture and incubated for 15 minutes to quench the reaction. Sep-Pak C18 Vac cartridges (Waters) were used for desalting. Equal amounts of labelled peptides were combined and then analyzed by LC-MS/MS as detailed below, and as previously described^45^.

For liquid chromatography−tandem mass spectrometry (LC−MS/MS) analysis, the TMT-labeled peptides were separated by a 120 min gradient elution at a flow rate 0.300 μL/min with the EASY-nLC 1000 system which was directly interfaced with a Thermo Orbitrap Fusion mass spectrometer. The analytical column was a ‘home-made’ fused silica capillary column (75 μm ID, 150 mm length; Upchurch, Oak Harbor, WA) packed with C-18 resin (300 A, 5 μm; Varian, Lexington, MA). Mobile phase A consisted of 0.1% formic acid, and mobile phase B consisted of 100% acetonitrile and 0.1% formic acid. The Orbitrap Fusion mass spectrometer was operated in the data-dependent acquisition mode using Xcalibur3.0 software. There was a single full-scan mass spectrum in the Orbitrap (350–1550 m/z, 120,000 resolution) followed by 3 seconds of data-dependent MS/MS scans in an ion routing multipole at 38% normalized collision energy. MS/MS spectra from each LC−MS/MS run were searched against the *Mycobacterium smegmatis* database using a Sequest HT algorithm in Proteome Discoverer software (PD, version 1.4). The search criteria were as follows: full tryptic specificity was required; two missed cleavages were allowed; oxidation (M) was set as a variable; carbamidomethylation (C) and TMTsixplex (K and N-terminal) were set as fixed modifications; precursor ion mass tolerance was set at 20 ppm for all MS and 20 mmu for all MS/MS spectra. The peptide false discovery rate (FDR) was estimated using percolator function provided by Proteome Discoverer (PD), and the cut-off score was accepted as 1% based on the decoy database. Relative protein quantification was performed using PD software according to the intensity of the TMT reporter ions. Peptides only assigned to a given protein group were considered as unique. Protein ratios were calculated as the median of all peptide hits belonging to a protein. Quantitative precision was expressed as protein ratio variability. Proteins with unique peptides >1 were included for analysis. The score of a certain protein in wild-type, *ΔhspX* or *hspX* over-expression strain was calculated according to the total score and the ratio between samples.

### Image acquisition, processing, and data analysis

Mycobacteria were centrifuged and resuspended in PBS buffer. For microscopy, 1~2 μl of the samples was spread over 1.4% agarose pads with a coverslip. Micrographs were obtained from living *M. smegmatis* cells using Delta Vision microscope (GE) with a 100x oil-immersion lens. Images were processed using FIJI (ImageJ) software. For the analysis, individual cells were picked manually (i.e. by the microscopist, not automated software) and data were extracted as fluorescence intensity per pixel along the cell lengths (or segments). All cells that could be confidently ascribed as individual cells within any chosen visual field were included in the analyses. Since cell lengths varies, the segment lengths were normalized and were displayed as a fractional distance. Quantification of pole and total cellular fluorescence intensity was calculated by the formula: ratio of fluorescence intensity (pole/total) = fluorescence of the region of interest (ROI) in pole-background ROI in pole/fluorescence of the region of interest (ROI) in total cell-background ROI in total cell, i.e. relative fluorescence of a region compared with average cell fluorescence. Statistical analysis of aggregation distribution and plotting of the data were performed with Excel (Microsoft) and R software. The number of bacilli analyzed is indicated in the figure legends. All experiments were performed at least 3 times independently on different days.

## Supplementary information

Supplementary Information

Table S1

Table S2
