# Supplementary material for: HspX promotes the polar localization of mycobacterial protein aggregates
Source: Sci Rep. 2019 Oct 10;9:14571. doi: 10.1038/s41598-019-51132-w (PMC6787098; doi:10.1038/s41598-019-51132-w)
Supplement: Supplementary file 1 — Supplementary Information [file 41598_2019_51132_MOESM1_ESM.pdf]

Title: HspX promotes the polar localization of mycobacterial protein aggregates

Authors: Yi-Wei Zhang[1], Jun-Hao Zhu[1,2,#], Zhen-Qi Wang[1], You Wu[1,3], Xianbin Meng[4], Xuhui Zheng[1,2] and Babak Javid[1*]

Affiliations:

[1] Centre for Global Health and Infectious Diseases, Collaborative Innovation Centre for the Diagnosis and Treatment of Infectious Diseases, Tsinghua University School of Medicine, Beijing, China

[2] School of Life Sciences, Peking University, Beijing, China

[3] College of Biological Sciences, China Agricultural University, Beijing, China

[4] MOE Key Laboratory of Bioinformatics, School of Life Sciences, Tsinghua University, Beijing, China

[#] present address: Immunology and Infectious Diseases, Harvard TH Chan School of Public Health, Boston, USA

YWZ and JHZ contributed equally to this work

[*] to whom correspondence should be addressed: bjavid@gmail.com

Supplementary Figure and Table Figure Legends

Fig. S1. GLR103 is an aggregation-prone protein in wild-type mycobacteria. (a) Wild-type *M. smegmatis* or *M. smegmatis*-Δ*hspX* expressing GLR or GLR103 proteins were lysed and subjected to differential ultra-centrifugation (see Methods), and the soluble, membrane (1% Triton-X-100 soluble) and insoluble (pellet) fractions subjected to SDS-PAGE followed by Western blotting with anti-GFP antibody. Anti-RpoB was used as a loading control. (b) Red fluorescence of the fractions as generated similarly to above was measured by fluorimetry.

Fig. S2. Deletion of *hspX* does not affect axenic growth of *M. smegmatis*. The growth rate (measured by OD_{600}) of wild-type *M. smegmatis* and *M. smegmatis*-Δ*hspX* or complemented by over-expression of *hspX* in complete 7H9 medium were compared. Measurements represent means +/- standard deviation of 3 biological replicates.

Fig. S3. Full-length HspX-GFP can complement *M. smegmatis*-Δ*hspX*. (a) Representative fluorescence images of red (GLR103) and green (HspX-GFP) channels of *M. smegmatis*-Δ*hspX*, expressing HspX-GFP and GLR103, with clear polar localization of the red fluorescence. Localization plots of both green (HspX) and red (GLR103) fluorescence of representative cells are shown below the micrographs. (b) The N-terminal deletion HspX cannot complement polar localization of GLR103.

Fig. S4. Western blot of HspX-GFP and ΔN35-HspX-GFP. Cells taken from experiment described in Fig. 2a. Blotting against RpoB was used as a loading control.

Fig. S5. The N-terminus of HspX is necessary but not sufficient for its polar localization. Fluorescence microscopy of *M. smegmatis*-Δ*hspX* expressing either full-length HspX-mCherry (HspX-mCherry), HspX-mCherry missing the N-terminal 35 amino acids (ΔN35-HspX-mCherry),

or the N-terminal 35 amino acids of HspX fused to mCherry (N35-mCherry) (top panel). Quantitative fluorescence measurements of several cells shown in the middle panel. The lower panel shows Western blot against mCherry or RpoB as loading control of sub-cellular fractions of the strains described above, verifying that only full-length HspX-mCherry is isolated from the insoluble fraction.

Fig. S6. SepF-GFP alone cannot redistribute GLR103 aggregates. (a) Green fluorescence channel of *M. smegmatis* expressing SepF-GFP and GLR103. (b) Brightfield, red and green channels of representative cells of the same strain. (c) Quantitation of aggregate localization in 60 representative cells of the above strain.

Fig. S7. Quantitative reverse-transcriptase PCR of *hspX* expression in wild-type and over-expressing cells.

Fig. S8. Quantitation of microscopy shown in Fig. 4.

Figure S9: Full-length Western blot from Fig. S1

Figure S10: Full-length Western blot from Fig. S4

Table S1. Proteins identified by spectrometry by tandem mass tags in *Mycobacterium smegmatis* aggregates

The abundance Score (see Methods) for proteins identified by mass spectrometry for *M. smegmatis* strains over-expressing *hspX* (OE), deleted for *hspX* (KO) and wild-type strain (WT). Proteins are in descending rank order for the OE strain.

Table S2. HspX-dependent aggregation-prone proteins

The relative abundance of proteins in aggregates in *M. smegmatis* over-expressing *hspX* (OE) compared with strains deleted for *hspX* (KO) and wild-type *M. smegmatis* (WT). Proteins are listed by descending order of ratio comparing OE/KO aggregates.

Figure S1

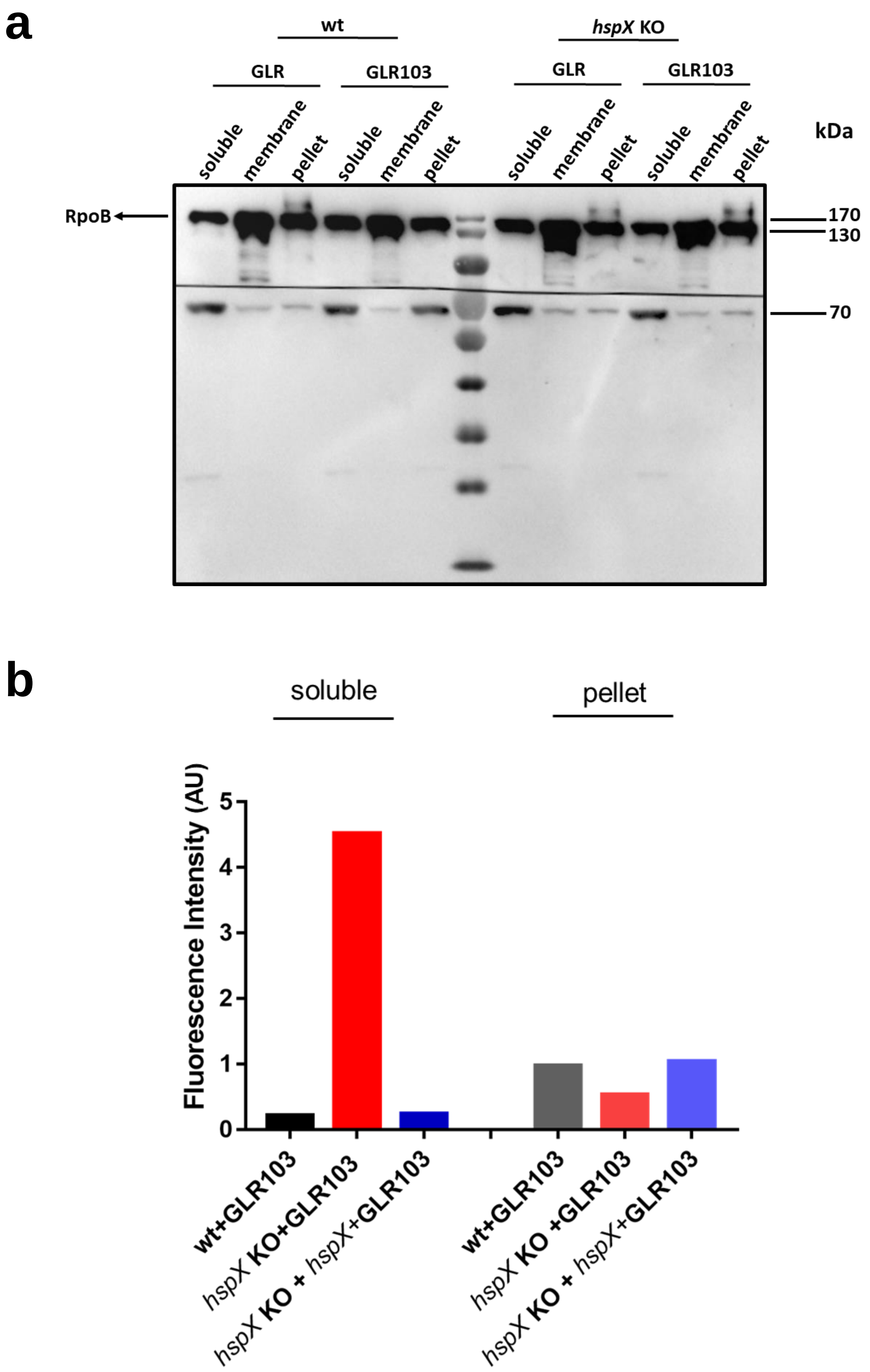
a
wt
hspX KO
GLR
GLR103
GLR
GLR103
soluble
membrane
pellet
soluble
membrane
pellet
soluble
membrane
pellet
soluble
membrane
pellet
kDa
RpoB
170
130
70
b
soluble
pellet
Fluorescence Intensity (AU)
5
4
3
2
1
0
wt+GLR103
hspX KO+GLR103
hspX KO + hspX+GLR103
wt+GLR103
hspX KO +GLR103
hspX KO + hspX+GLR103

Figure S2

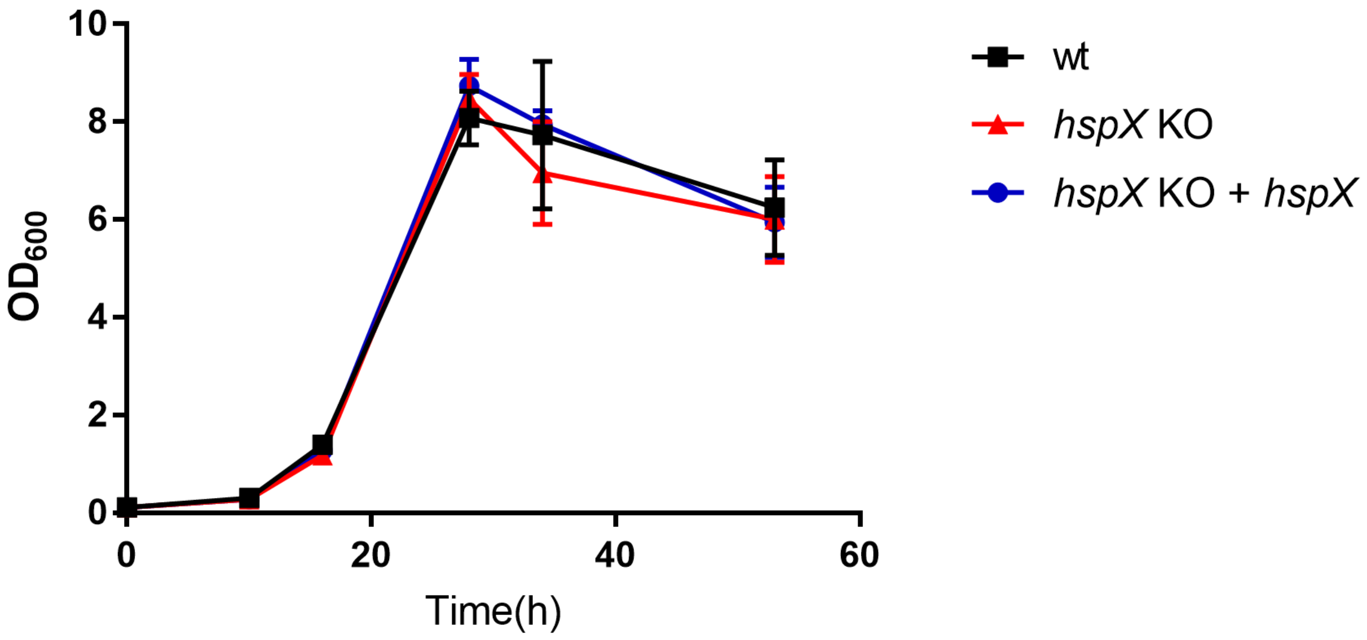

Figure S3

a

RFP **GFP** **merge**

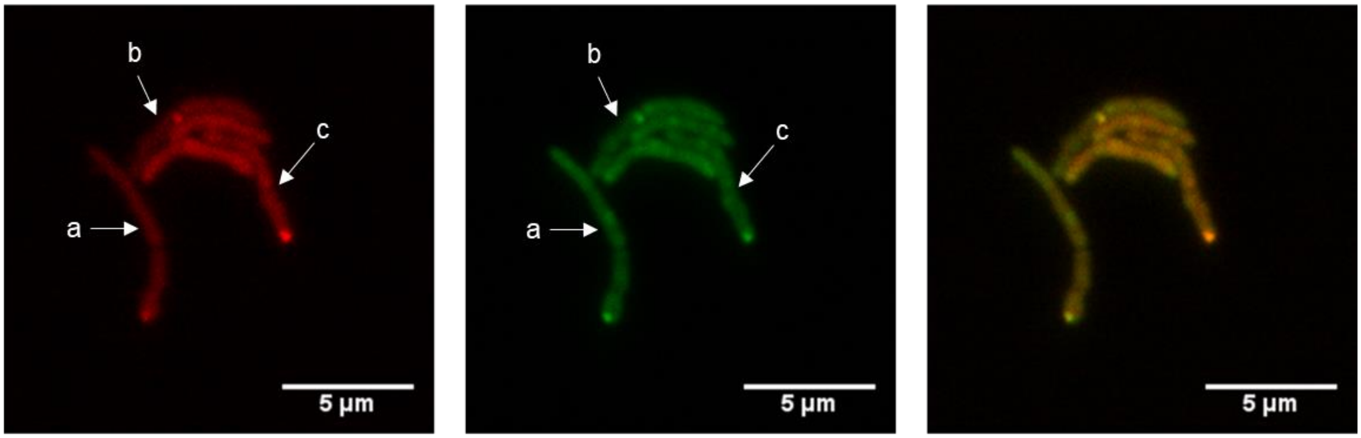

GLR103 + HspX-GFP

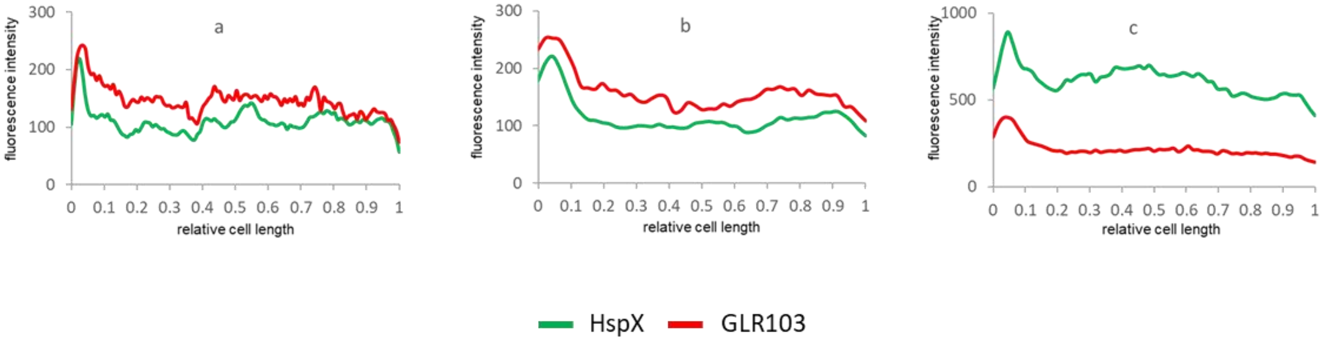

b

RFP

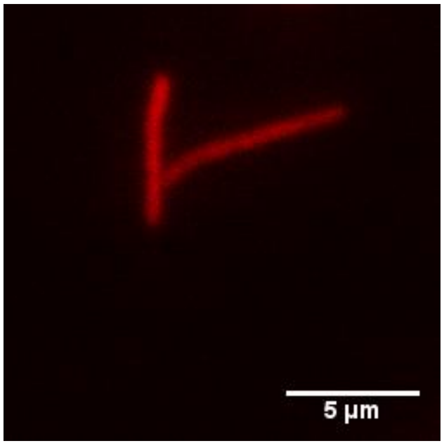

GLR103 + ΔN-HspX

Figure S4

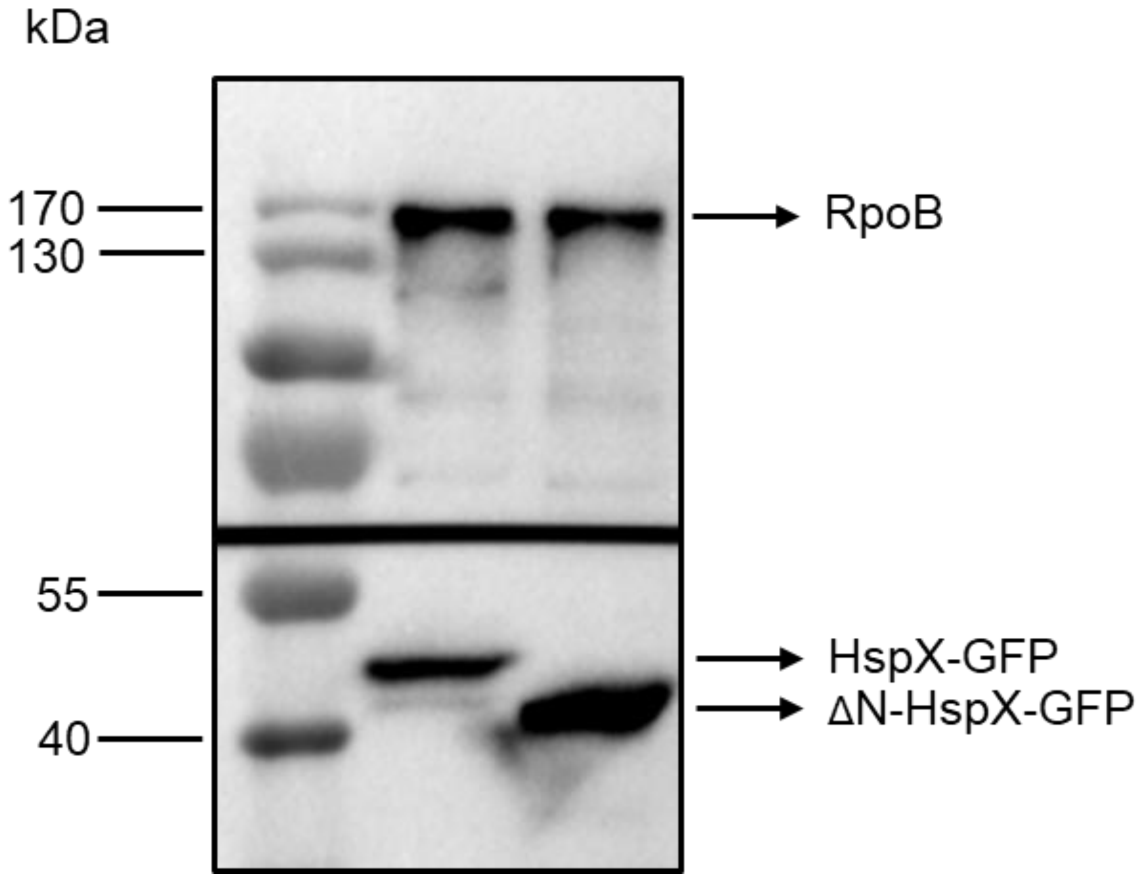

Figure S5

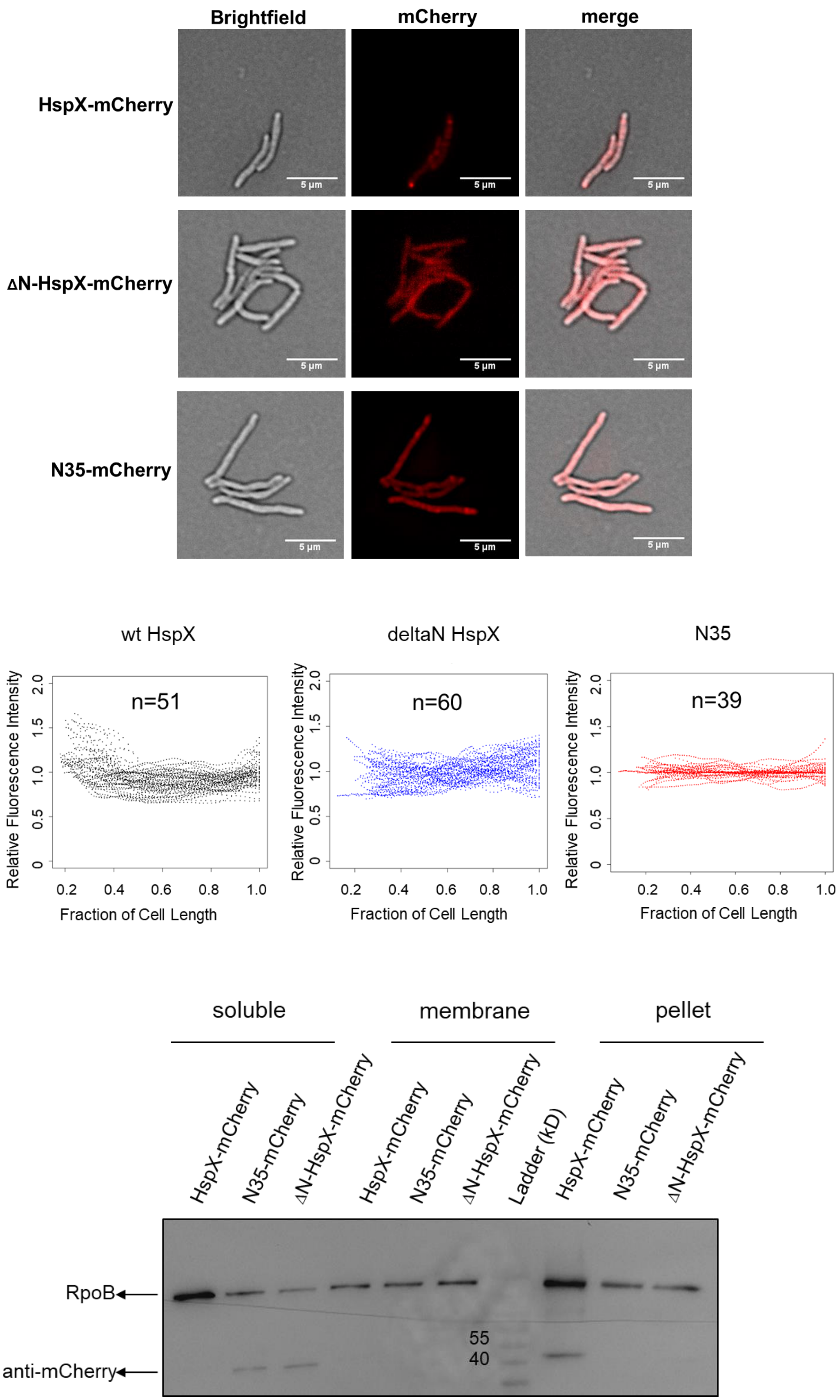

Brightfield
mCherry
merge
HspX-mCherry
ΔN-HspX-mCherry
N35-mCherry
5 µm
wt HspX
deltaN HspX
N35
n=51
n=60
n=39
Relative Fluorescence Intensity
Fraction of Cell Length
0
0.5
1.0
1.5
2.0
0.2
0.4
0.6
0.8
1.0
soluble
membrane
pellet
HspX-mCherry
N35-mCherry
ΔN-HspX-mCherry
Ladder (kD)
RpoB
anti-mCherry
55
40

Figure S6

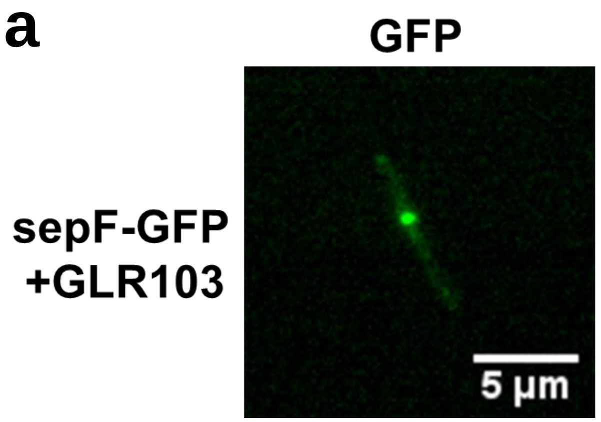

a
GFP
sepF-GFP
+GLR103
5 µm

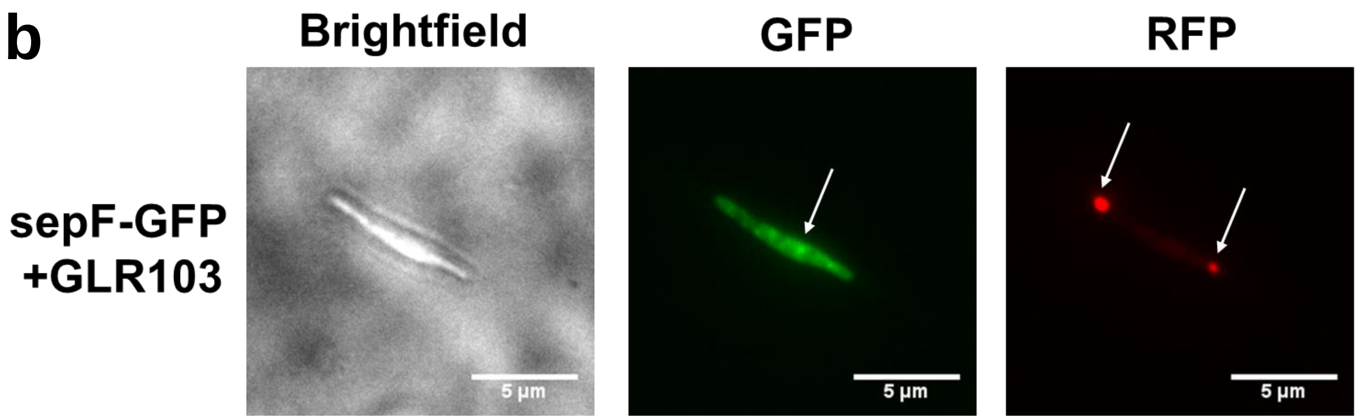

b
Brightfield
GFP
RFP
sepF-GFP
+GLR103
5 µm
5 µm
5 µm

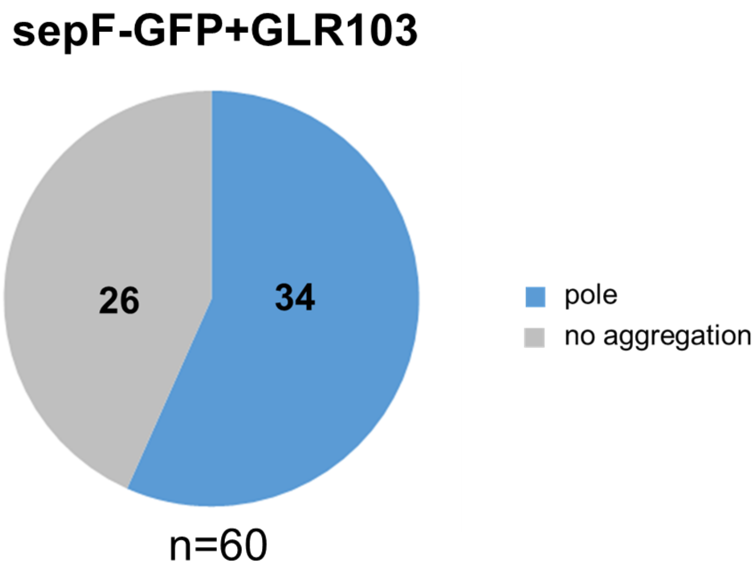

sepF-GFP+GLR103
26
34
pole
no aggregation
n=60

Figure S7

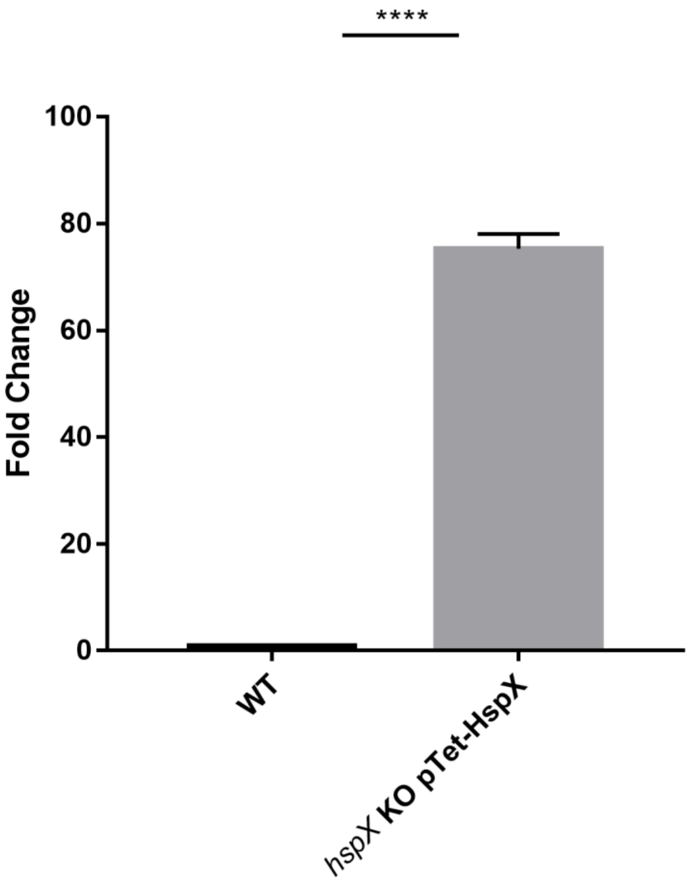

Figure S8

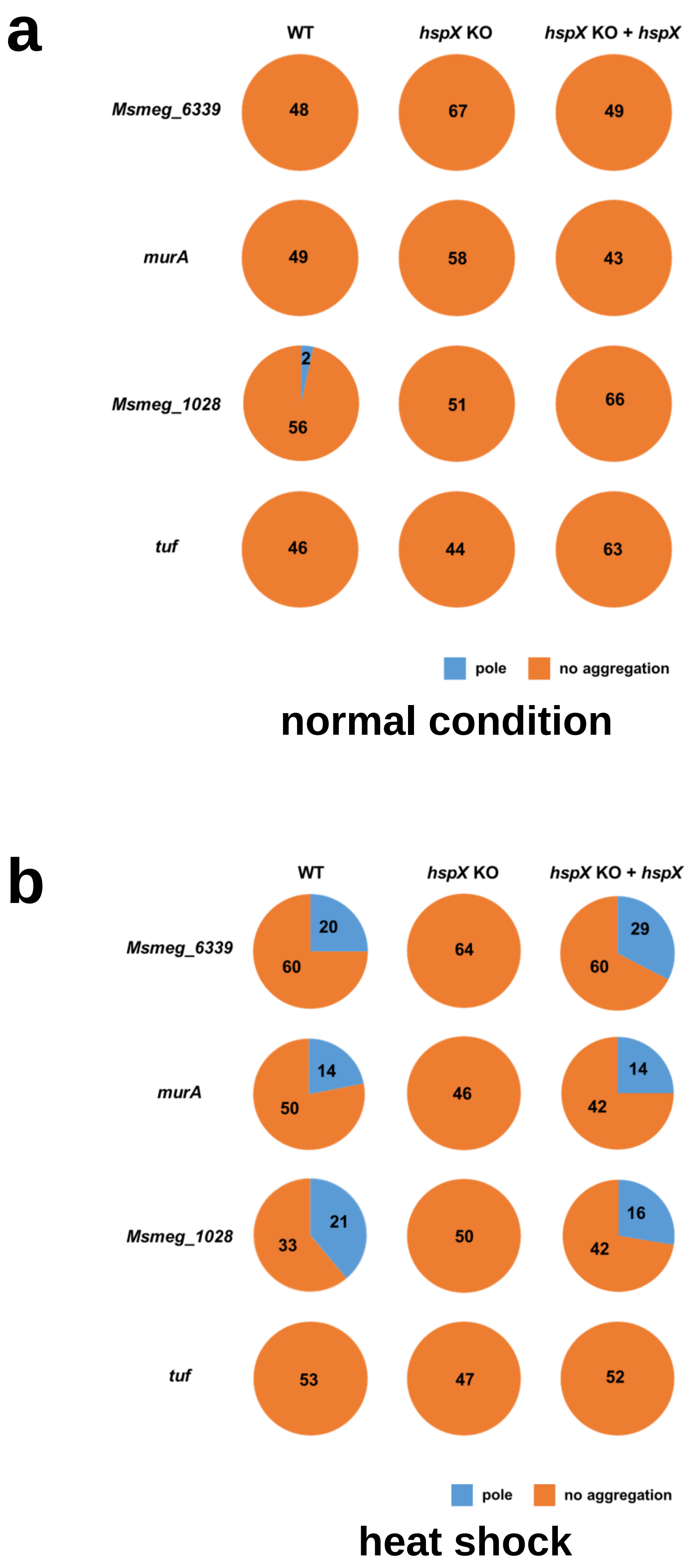
a
WT
hspX KO
hspX KO + hspX
Msmeg_6339
48
67
49
murA
49
58
43
Msmeg_1028
2
56
51
66
tuf
46
44
63
pole
no aggregation
normal condition
b
WT
hspX KO
hspX KO + hspX
Msmeg_6339
20
60
64
29
60
murA
14
50
46
14
42
Msmeg_1028
21
33
50
16
42
tuf
53
47
52
pole
no aggregation
heat shock

Figure S9

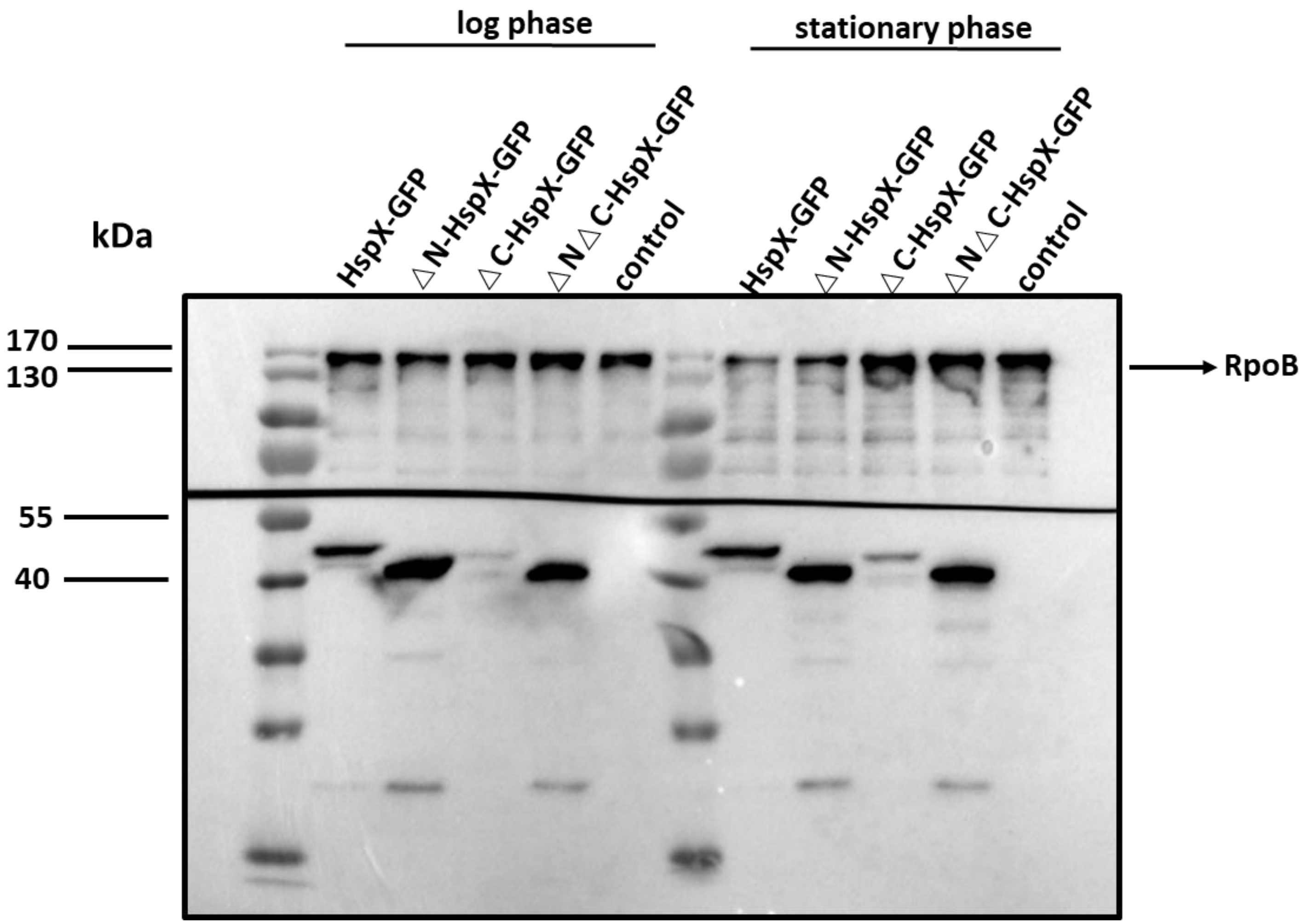
log phase
stationary phase
kDa
HspX-GFP
△N-HspX-GFP
△C-HspX-GFP
△N△C-HspX-GFP
control
HspX-GFP
△N-HspX-GFP
△C-HspX-GFP
△N△C-HspX-GFP
control
170
130
55
40
RpoB

Figure S10

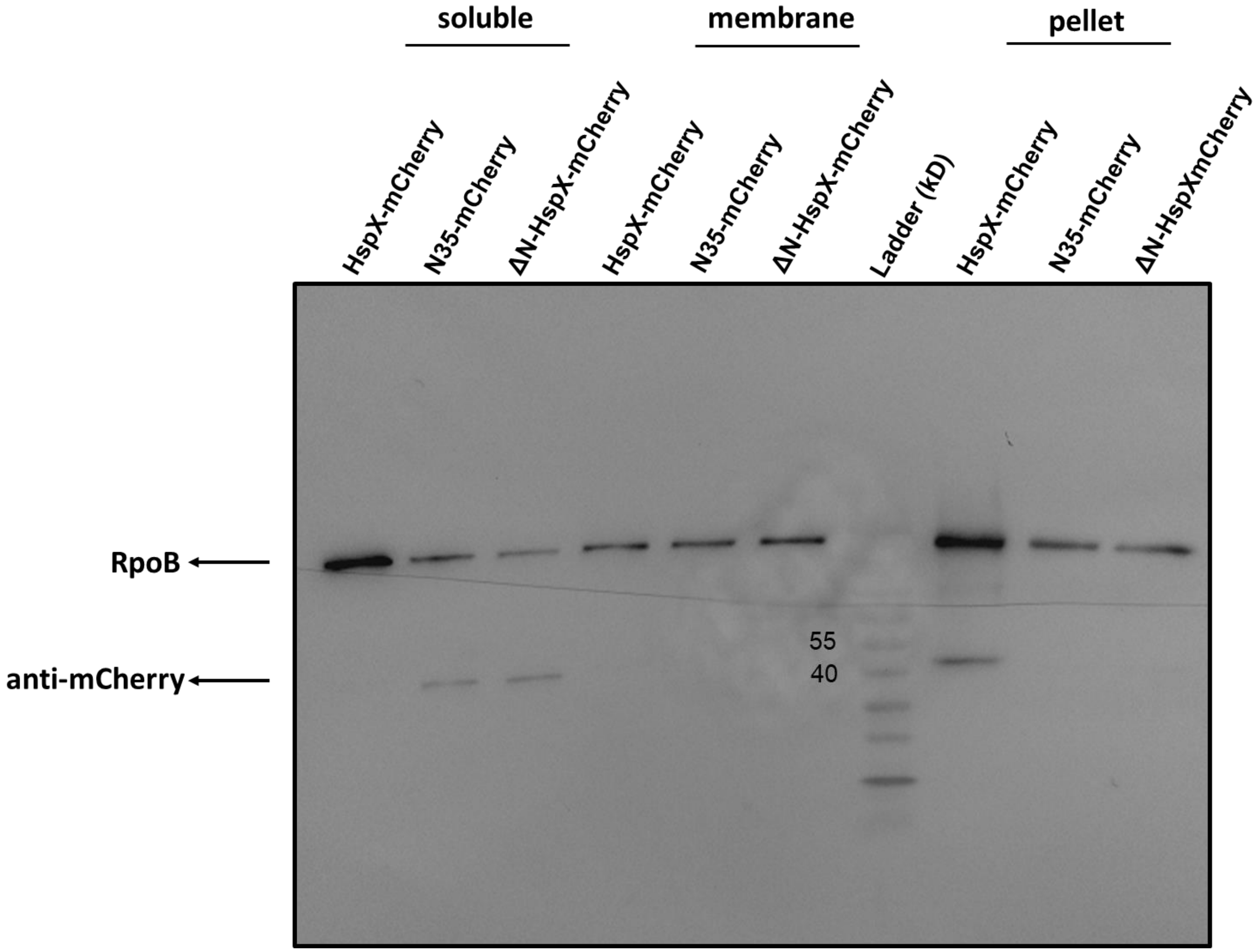
soluble
membrane
pellet
HspX-mCherry
N35-mCherry
ΔN-HspX-mCherry
HspX-mCherry
N35-mCherry
ΔN-HspX-mCherry
Ladder (kD)
HspX-mCherry
N35-mCherry
ΔN-HspXmCherry
RpoB
anti-mCherry
55
40
